# ENGAGE: Analyzing the value of virtual reality in a patient-centric immersive learning program in myasthenia gravis for healthcare professionals

**DOI:** 10.3389/fneur.2025.1655351

**Published:** 2026-01-13

**Authors:** Ina Weisshardt, Cornelia Reyes Acosta, Trishna Chauhan, Kaloyan Haralampiev, Andrijana Mušura Gabor, Alexis Rodriguez, Allison Foss, Ashwin Pinto, Channa Hewamadduma, John Vissing, Nicholas J. Silvestri, Sophie Lehnerer, Marc De Backer, Natasha Monin, Sophie Barry, Ivo Vlaev

**Affiliations:** 1 LLH Concepts GbR, Haimhausen, Germany; 2 The University of Liverpool, Liverpool, United Kingdom; 3 Sofia University, Sofia, Bulgaria; 4 Behavioral and Experimental Economics Lab, Zagreb School of Economics and Management, Zagreb, Croatia; 5 MG Georgia, Atlanta, GA, United States; 6 Myasthenia Gravis Association (MGA), Kansas City, MO, United States; 7 Division of Clinical Neuroscience, University of Southampton, Southampton, United Kingdom; 8 Academic Neuromuscular Unit, Sheffield Teaching Hospitals NHS Foundation Trust, Sheffield, United Kingdom; 9 Sheffield Institute for Translational Neuroscience (SITraN), University of Sheffield, Sheffield, United Kingdom; 10 Department of Neurology, Copenhagen Neuromuscular Center, Rigshospitalet, University of Copenhagen, Copenhagen, Denmark; 11 Department of Neurology, Jacobs School of Medicine and Biomedical Sciences, University at Buffalo, Buffalo, NY, United States; 12 Department of Neurology and Experimental Neurology, Charité – Universitätsmedizin Berlin, Berlin, Germany; 13 UCB, Brussels, Belgium; 14 UCB, Malvern, VIC, Australia; 15 UCB, Dublin, Ireland; 16 Centre for Behavioral and Implementation Science Interventions (BISI), Yong Loo Lin School of Medicine, National University of Singapore, Singapore

**Keywords:** Myasthenia gravis, patient–healthcare professional communication, virtual reality, immersive learning, shared decision-making

## Abstract

**Background:**

Myasthenia gravis (MG) is a chronic autoimmune neuromuscular disease characterized by muscle weakness that can significantly impact patients’ lives. Recent patient-led research highlighted a disconnect between healthcare professionals (HCPs) and patients, emphasizing the need for effective and empathetic patient–HCP dialogue and shared decision-making (SDM). The power of virtual reality (VR) to increase empathy and provide impactful learning experiences has been established. This outcome evaluation assessed the ability of VR to improve HCPs’ knowledge, attitudes and empathy in MG, aiming to strengthen patient–HCP communication and facilitate SDM.

**Methods:**

The ENGAGE educational pilot program comprised a needs assessment, a VR-based intervention and an outcome evaluation. Content for the VR intervention was developed using a patient-centric approach integrating patient and HCP voices. The VR module simulated “a day in the life of Julia,” a virtual patient with MG, allowing HCPs to experience MG symptoms and their impact on a patient’s life. The experience was implemented in hospital-based workshops. The outcome evaluation included surveys assessing Moore’s Levels 2–4, the Theoretical Domains Framework (TDF), and SDM, and was supplemented by semi-structured interviews. Quantitative and qualitative data were analyzed using SPSS Statistics and thematic analysis, respectively.

**Results:**

Eighty-seven HCPs completed the VR experience across 12 workshops. Sixty HCPs participated in the outcome evaluation survey, and 10 participated in interviews. HCPs reported high satisfaction with the immersive learning, citing its relevance and ease of use. Based on survey responses, HCPs’ most important learnings were a “better understanding of the impact of MG on patients’ lives” (*n/N* = 46/60) and “developing empathy for how a patient with MG might feel” (*n/N* = 37/60). HCPs expressed commitment to changing their practice. Quantitative analysis revealed significant improvements in most TDF domains and SDM post-intervention, with the TDF domain “beliefs about capabilities” emerging as the strongest predictor of SDM.

**Conclusion:**

Our study found that the immersive VR intervention effectively increased HCP empathy, knowledge and attitudes in MG care. The program’s patient-centric design ensured content relevance. These findings suggest that VR-based learning is a valuable tool for medical education and the improvement of SDM, particularly in rare diseases like MG.

## 1 Introduction

Myasthenia gravis (MG) is a rare neuromuscular disease characterized by muscle weakness and fatigue, resulting from the production of autoantibodies targeting the neuromuscular junction (1, 2). The heterogeneous and unpredictable nature of MG presents challenges, including fluctuations in symptoms, potential disease exacerbations and differing response to treatment, that profoundly affect patients’ daily lives (1–4). A patient-led analysis revealed the lived experience of MG from the patient’s perspective. Five overarching themes were identified that describe the reality of living with MG. These themes included living with fluctuating and unpredictable symptoms; a constant state of adaptation; treatment inertia; a sense of disconnect with healthcare professionals (HCPs); and feelings of anxiety, frustration, guilt, anger, loneliness and depression (5). The identified sense of disconnect between HCPs and patients emphasizes the need for effective and empathetic patient–HCP dialogue and engagement in shared decision-making (SDM). SDM is a joint process in which HCPs and patients work together to reach a decision about care (6). It is essential to empower patients as partners in their own care (7, 8).

In this context of unmet patient needs and the lack of standards for SDM (7), especially in the field of rare diseases, we set out to design a research-based innovative immersive learning program for HCPs to improve patient–HCP interaction and communication. Immersive learning tools, such as virtual reality (VR) and simulation-based training, provide interactive experiences that engage individuals’ emotions and enhance memory retention, creating impactful and lasting learning outcomes (9). The role of immersive learning in educating HCPs to drive behavioral change through better awareness and understanding of the impact of a disease on a patient’s life has previously been described (10). Furthermore, the power of VR to enhance empathy has recently been explored in a research paper by Dhiman (11). When immersed in realistic, virtual environments, HCPs are better able to appreciate patients’ needs and are primed to make decisions aligned with patients’ values and preferences (11). A recent study utilizing VR showed significant increases in knowledge, attitudes and empathy toward patients suffering from psychosis; this effect was found to be particularly strong among participants in younger age cohorts (12).

We designed the ENGAGE program to enhance HCPs’ knowledge, attitudes and empathy in MG in order to trigger behavioral change. Through use of an immersive VR experience, we aimed to enhance patient–HCP communication and facilitate SDM in MG to improve patients’ outcomes. We chose to utilize VR technology in ENGAGE for its ability to enhance learners’ empathy toward patients living with MG through ‘hands-on’ engagement with the subject matter, thus allowing learners to experience new information (13). The program was designed to target the unmet needs and lack of standards for SDM, especially in the field of rare diseases (14).

This paper explores the impact of this immersive educational activity on HCPs by addressing the questions, “To what extent does a VR-assisted immersive learning experience enhance HCPs’ knowledge, attitudes and empathy toward patients living with MG, their symptoms, and the impact of MG on their day-to-day lives?” and “Does VR-assisted immersive learning change HCP behavior in relation to SDM?”

## 2 Methods

### 2.1 Study design and setting

**Figure 1:**
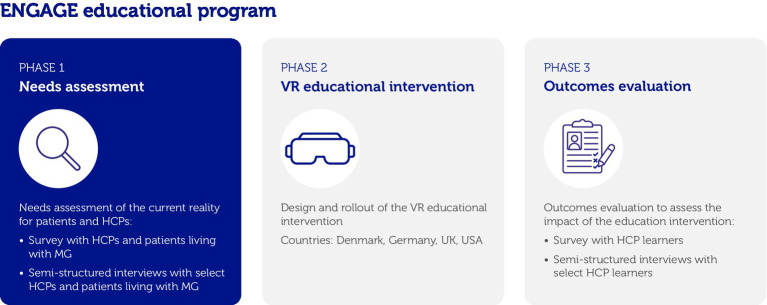
Study design of ENGAGE. HCP, healthcare professional; MG, myasthenia gravis; UK, United Kingdom; USA, United States of America; VR, virtual reality. Figure reproduced with permission from Reyes Acosta et al. Copyright © 2025. The Author(s). (15).

The ENGAGE program was a three-phase medical education program, comprising a needs assessment (Phase 1) that included patient and HCP voices to define the educational content (15), a VR intervention (Phase 2) to realize the educational content, and an outcome evaluation (Phase 3; Figure 1).

A collaborative, patient-centric approach engaging various stakeholders was used in the design process, as this has been reported to be beneficial for information exchange processes in practice in rare diseases (16). This approach was also reflected in the setup of the steering committee, which consisted of four people living with MG, some of whom were representatives of patient organizations, and five HCPs who were experts in the management of MG. The steering committee was led by the program developer and facilitator (LLH Concepts) and included one representative from the sponsor (UCB) to reflect the collaborative approach chosen for this project. The sponsor did not have direct access to the collected data and was not directly involved in the conduct of the analysis. Primary data were only available to essential members of the research team at LLH Concepts unless explicit consent was received from the respective participant.

### 2.2 Development of the VR module

The VR module consisted of a virtual patient case titled *“A day in the life of Julia, a young woman with undiagnosed MG.”* Three scenarios in Julia’s daily life—at home, at work and at a physician’s office—were developed for the learner by a technical service provider (Berlin, Germany). All situations in the VR module mirrored situations that were reported by patients in the needs assessment (15). To ensure the VR module accurately represented the patient experience, it was reviewed and tested by the patient members of the steering committee. To maximize the immersive experience, the VR device consisted of a VR headset and two handset devices for the execution of specific motor tasks. Wearing the VR headset and using the handset devices, HCPs saw and actively interacted within a computer-generated world; they virtually experienced life with MG, facing everyday challenges from the patient’s perspective, including walking around, executing motor tasks, interacting and perceiving the world through the patient’s eyes. Over the course of the module, HCPs experienced various symptoms of MG (ptosis, double vision, muscle weakness and fatigue) and fluctuations of these symptoms. The choice of symptoms was driven by input from patients and HCPs during the needs assessment, by insights from patients on the steering committee, and by the functionalities and limitations of the technology used. Scenarios allowed users to experience the impact of the chosen symptoms—for example, limitations in their ability to work, and the reactions of others in social settings. The last scenario was a patient–HCP dialogue enabling HCPs to experience this from a patient’s perspective.

### 2.3 Implementation of the VR module

Twelve VR workshops were conducted with HCPs at hospitals in Denmark, Germany, the United Kingdom and the United States. Invitations to participate were circulated to each hospital’s head of staff by members of the steering committee. Participation was open to all HCPs; there were no inclusion or exclusion criteria, nor predetermined recruitment proportions based on specialization. The workshops were run and moderated by representatives of LLH Concepts and the technical service provider. VR headsets and handset devices to access the VR module were provided by the organizers. Following the VR immersion, all HCP participants were invited to share their experience in a group debrief session and to fill out the outcome evaluation survey and/or to participate in an interview.

### 2.4 Needs assessment and outcome evaluation

The needs assessment and outcome evaluation questionnaires were delivered via Qualtrics.^1^ In accordance with the study design, HCPs completed both the needs assessment and outcome evaluation, and patients only completed the needs assessment. The questionnaires combined various frameworks that determine the needs and outcomes of educational projects and linked these with recommended interventions. The HCP questionnaires consisted of demographic data and questions to assess Moore’s Levels 2–4 (self-reported by HCPs) and Theoretical Domains Framework (TDF) items, with an SDM inventory tailored specifically to HCPs working in MG. Explanations of Moore’s framework and the TDF are provided below.

The questionnaire in the outcome evaluation was used to assess changes in HCPs’ knowledge and attitudes using survey items from the TDF/Capability, Opportunity, Motivation and Behavior (COM-B) model and the educational outcome level defined by Moore’s model evaluation framework (17). We focused on the first four levels of Moore’s model [1. Participation; 2. Satisfaction; 3. Learning; and 4. Competence (knowledge and attitudes)], with Moore’s Level 1 assessed through participation in the VR experience itself.

Questionnaires were complemented by semi-structured interviews with patients and HCPs. In the outcome evaluation interviews, HCPs were asked about their experiences with VR, how their knowledge, attitudes and empathy toward patients with MG had changed following their training, and whether they planned to change their clinical practice.

### 2.5 Moore’s framework

To address the gap in professional practice and the shortcomings of continuing medical education (CME), Moore et al. (18) developed a framework outlining stages of clinician learning and its impact on patient outcomes. This framework includes seven outcome levels: 1. Participation; 2. Satisfaction; 3. Learning; 4. Competence; 5. Performance; 6. Patient health; and 7. Population health. Moore’s framework is widely recognized and commonly applied in CME. While it can aid in planning educational activities, it is primarily used to assess outcomes in continuing education for HCPs (19).

The most recent framework suggests three tasks for evaluating the effectiveness of a learning activity. These are assessing learning (summative assessment), assessing changes in learner performance (performance assessment) and assessing changes in patient health (impact assessment) (19). Due to challenges in collecting objective data, the framework allows flexibility in choosing outcome measures, including both objective observations and self-reports (19).

### 2.6 The Theoretical Domains Framework

The TDF synthesizes concepts from numerous psychological theories to identify factors influencing behavior change (behavioral barriers and enablers) (17), aiding in areas like SDM. The TDF is used together with the COM-B model, which details that behavior change requires capability, opportunity, and motivation. The COM-B model serves as a concise and overarching framework for understanding the mechanisms behind behavior and for developing targeted interventions that lead to effective behavioral change. TDF domains can be conceptualized and categorized across the three COM-B components: capabilities, opportunities and motivations. Building on the COM-B model, the Behavior Change Wheel provides a standardized framework for designing effective interventions and suggests specific intervention functions that are most appropriate for targeting identified barriers (17, 20, 21).

### 2.7 Data analysis

Quantitative and qualitative data were analyzed separately. Qualitative data from the interviews were analyzed using Braun and Clarke’s thematic analysis framework (22). For the outcome evaluation, focus was placed on how the VR immersive learning impacted HCPs’ knowledge, their appreciation of symptoms and impact on life for patients with MG, and their attitude toward change. We then deductively mapped this onto Moore’s framework.

Quantitative survey data were collected via Qualtrics (see text footnote 1), downloaded from the platform and then analyzed using SPSS Statistics 26.0. Data cleaning consisted of removing responses with more than 50% of the data missing. Survey responses were compared pre-intervention (needs assessment) and post-intervention (outcome evaluation).

A quantitative analysis using the TDF was carried out to statistically compare domains mapped to capabilities, opportunities and motivations in relation to patient–HCP dialogue and SDM before and after the immersive learning experience. After the coding of TDF was reversed for relevant items, Cronbach’s alpha was calculated for each domain. Domains with alpha <0.7 were dropped from the analysis, and where relevant, items were separated and renamed (optimism, beliefs about consequences, goals, memory and attention, and emotion). Descriptive statistics were calculated for demographic data, the composite measure of SDM and for the TDF domains and items. Pre- and post-intervention comparisons of TDF domains mapped to capabilities, opportunities, and motivations were conducted using independent samples analysis. Comparison of paired (repeated) samples was not possible due to the use of de-identified participant data, which prevented the formation of pairs. Shapiro–Wilk and Kolmogorov–Smirnov tests of normality were conducted to determine whether results on TDF domains and SDM were normally distributed. If they were not, it was planned to employ the Mann–Whitney nonparametric test to establish the statistical significance of the intervention on SDM. In addition to statistical significance, the magnitude of the observed change was quantified using effect sizes and explained variance. For the nonparametric comparisons, the standardized effect size (r) was derived from the z values of the Mann–Whitney U test (*r* = z /√N). The construct variable of SDM was regressed on the measures of TDF domains using a stepwise multiple regression. This determined whether different TDF domains predicted SDM, and which behavioral interventions are deemed appropriate.

## 3 Results

Full results from the needs assessment have been published separately (15). This publication presents results from the outcome evaluation. For quotes from participants, supporting the qualitative insights, please refer to Supplementary Table 1.

### 3.1 Participation (Moore’s Level 1)

A total of 133 patients and 55 HCPs participated in the needs assessment (Phase 1), of whom 122 patients and 47 HCPs completed the needs assessment survey; 10 patients and 10 HCPs participated in the associated interviews. Eighty-seven HCPs completed the VR experience (Phase 2) across 12 workshops. Sixty HCPs participated in the outcome evaluation (Phase 3) survey, and 10 HCPs participated in the associated interviews (Table 1). The HCPs who participated in the needs assessment did not necessarily participate in the outcome evaluation, and vice versa. Not all HCPs who completed the needs assessment could attend the VR experience.

**Table 1:** Distribution of questionnaires and interviews among HCPs and patients.

Phase	Method	Participated		Completed	
		HCPs	Patients	HCPs	Patients
Needs assessment	Survey (Part 1)	*N* = 55	*N* = 133	*N* = 47	*N* = 122
	Interview	*N* = 10	*N* = 10	*N* = 10	*N* = 10
Outcome evaluation	Survey (Part 2)	*N* = 60		*N* = 60	
	Interview	*N* = 10		*N* = 10	

HCP, healthcare professional.

### 3.2 Sample demographics

The demographics of HCPs who had available data following participation in the needs assessment (Phase 1) and outcome evaluation (Phase 3) surveys are described in Table 2. Survey responses with incomplete answers were removed for evaluation of TDF data. After data cleaning, there were 45 responses from HCPs included from the needs assessment and 55 from the outcome evaluation. Of the HCPs who participated in the outcome evaluation, 50.9% (*n* = 28/55) were neurologists or neuromuscular specialists and 10.9% (*n* = 6/55) were nurses, including specialist nurses.

**Table 2:** HCP participant demographics.

Category	Needs assessment (*N* = 47)	Outcome evaluation (*N* = 55)
Gender		
Male	17 (36.2%)	14 (25.5%)
Female	28 (59.6%)	39 (70.9%)
Other	2 (4.3%)	2 (3.6%)
Age, mean (SD)	39.7 (9.7)	40.3 (13.4)
Years of experience		
<1 year	5 (10.6%)	13 (23.6%)
1–2 years	15 (31.9%)	11 (20.0%)
3–5 years	13 (27.7%)	12 (21.8%)
5–10 years	3 (6.4%)	2 (3.6%)
10–15 years	6 (12.8%)	8 (14.5%)
>15 years	5 (10.6%)	9 (16.4%)
Country of work		
USA	14 (29.8%)	22 (40.0%)
UK	13 (27.7%)	13 (23.6%)
Denmark	7 (14.9%)	5 (9.1%)
Germany	13 (27.7%)	14 (25.5%)
Workplace		
Solo practice	1 (2.1%)	1 (1.8%)
Group practice	2 (4.3%)	3 (5.5%)
Multidisciplinary Healthcare center	8 (17.0%)	11 (20.0%)
Hospital clinic	35 (74.5%)	36 (65.5%)
Other	1 (2.1%)	4 (7.3%)
Job category		
Neurology	13 (27.7%)	14 (25.5%)
Neuromuscular specialist	15 (31.9%)	14 (25.5%)
General practitioner	0 (0%)	1 (1.8%)
Medical student/Residential year/PJ	0 (0%)	0 (0%)
Nurse/specialist nurse	5 (10.6%)	6 (10.9%)
Physiotherapist	1 (2.1%)	3 (5.5%)
Alternative practitioner	5 (10.6%)	10 (18.2%)
Other	8 (17.0%)	7 (12.7%)

HCP, healthcare professional; PJ, prospective doctor in the practical year; SD, standard deviation; USA, United States of America; UK, United Kingdom.

### 3.3 Satisfaction (Moore’s Level 2)

HCPs rated their overall experience of the learning activity positively, with a mean rating of 4.44 [standard deviation (SD) 0.66; *n* = 54] out of 5.00 on a scale of 1 (“very poor”) to 5 (“excellent”). No participants rated the experience less than 3 (“neutral”). Across the post-intervention survey and interviews, satisfaction with the activity was largely related to relevance of the content to daily practices and ease of engagement. Content relevance achieved a mean rating of 4.49 (SD 0.74; *n* = 53) out of 5.00; minimum: 2.00, maximum: 5.00, on a scale of 1 (“strongly disagree”) to 5 (“strongly agree”), and HCPs described the VR technology as easy to use and understand (Supplementary Table 1). Overall, interviews corroborated the quantitative survey results – HCPs found the VR-based activity “*immersive*,” “*insightful*” and effective as a learning tool, and were satisfied with the duration and level of technology used. Some HCPs even suggested superiority of the experience to traditional textbooks: “…*that’s far better than reading it in a textbook*,” “y*ou do not remember what you read in a book, but you remember a VR experience*” (Supplementary Table 1).

### 3.4 Knowledge gain and changes in attitudes and empathy (Moore’s Level 3a, subjective)

**Figure 2:**
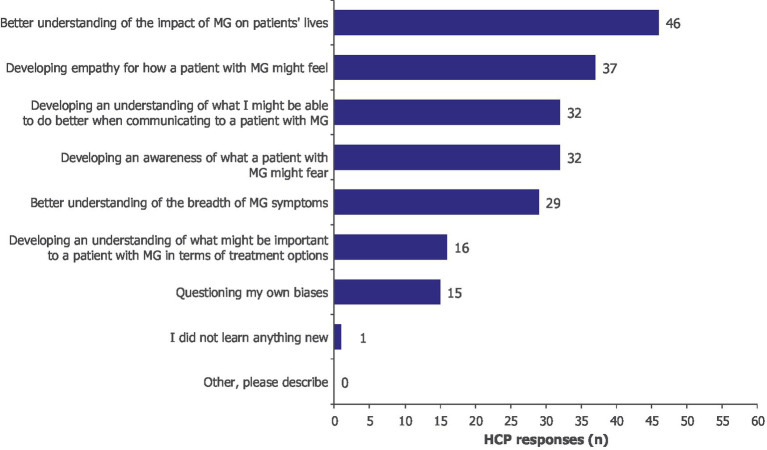
What was the most important learning for you? Moore’s Level 3a, subjective. HCPs could select more than one option; 60 HCPs provided 208 responses. HCP, healthcare professional; MG, myasthenia gravis.

The VR experience increased the declarative knowledge of HCPs, which in turn facilitated the development of empathy toward patients with MG (Supplementary Table 1). Indeed, after participating in the VR experience, HCPs reported a better understanding of the breadth of MG symptoms (*n/N* = 29/60), including their overall effects and fluctuations, and increased empathy toward patients with MG (*n/N* = 37/60). The most important learning was a better understanding of the impact of MG on patients’ everyday lives (*n/N* = 46/60; Figure 2).

Interviews mirrored the survey results, with HCPs communicating an increased understanding of symptoms such as ptosis and diplopia, which in turn prompted them to “*…take a different perspective and empathize a little bit more with patients.*” Some HCPs indicated that the program allowed them to see beyond the physical symptoms of MG, to consequences such as frustration, explaining *“…the VR experience really heightened that frustration element…*.” Furthermore, opportunities to enhance communication with their patients and the multidisciplinary team were recognized. Enhanced empathy motivated HCPs to improve communication skills and advocate for tailored treatments (Supplementary Table 1).

### 3.5 Procedural knowledge gain (Moore’s Level 3b, subjective)

**Figure 3:**
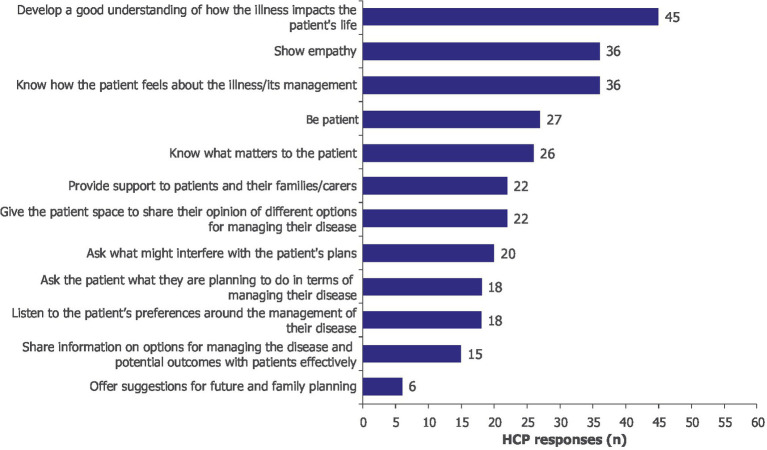
After participating in the learning activity, I am better prepared to… Moore’s Level 3b, subjective. HCPs could select more than one option; 60 HCPs provided 291 responses. HCP, healthcare professional.

Procedural knowledge gain was identified as an important element for enhancing SDM. Increased understanding of how MG impacts patients’ lives (*n/N* = 45/60), confidence in the ability to empathize with patients (*n/N* = 36/60), and an improved understanding of what matters to patients were reported by HCPs (*n/N* = 26/60) (Figure 3). After participating in the learning activity, HCPs gained confidence in understanding patients beyond clinical symptoms, which motivated them to take a more patient-centered approach to SDM. Qualitative accounts supported the survey results and described shifts in clinical practice; HCPs suggested that they would reframe questions toward “*symptoms in the context of one’s day-to-day life*” to “*understand what the lived experience is with this disease*” (Supplementary Table 1).

### 3.6 Self-reported competence gain and commitment to change (Moore’s Level 4)

**Figure 4:**
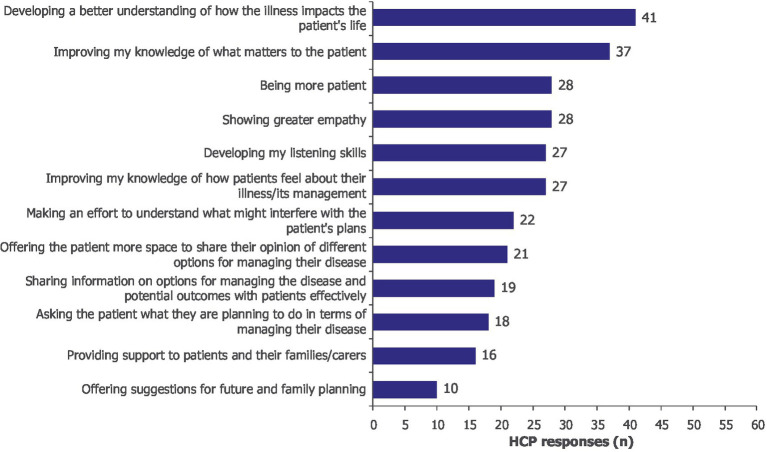
As a result of this learning activity, I will likely make changes to my practice by… Moore’s Level 4. HCPs could select more than one option; 60 HCPs provided 294 responses. HCP, healthcare professional.

Participants expressed a commitment to changing their practice following the immersive learning experience. This included developing a better understanding of how the illness impacts patients’ lives (*n/N* = 41/60), improving knowledge of what matters to patients (*n/N* = 37/60), increasing patience (*n/N* = 28/60), showing greater empathy (*n/N* = 28/60) and developing listening skills (*n/N* = 27/60; Figure 4).

Competence gain and commitment to change were also captured qualitatively in the outcome evaluation interviews (Supplementary Table 1). One HCP (a nurse specialized in MG) shared a recent example of how the immersive learning experience increased their awareness of SDM and how they engaged with their patients regarding treatment decisions: “*If a patient says ‘No, I do not want treatment’, to say ‘Actually, why don’t you? What’s that all about? What’s stopping you? Yeah, what are your concerns?*’” HCPs were enthusiastic about integrating the insights from their VR experience into their practice. Participants also showed intent to share their learnings with fellow team members to improve the training of junior team members and non-specialists. Additionally, the experience was seen as beneficial for fostering better communication within healthcare teams, ensuring that all members have a similar understanding of what it is like to live with MG, thus promoting more cohesive and patient-centered care.

### 3.7 Impact on SDM, capabilities, opportunities and motivations

Owing to the lack of normally distributed variables (Supplementary Table 2), statistical significance between pre- and post-intervention SDM and TDF domains was determined with the Mann–Whitney U test and, due to small sample sizes, verified by Monte Carlo simulation (Supplementary Table 3).

**Figure 5:**
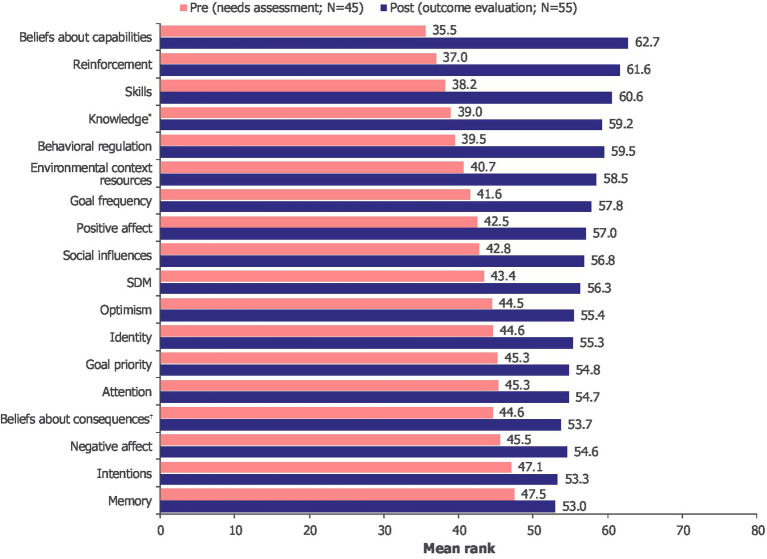
Changes in mean rank pre-intervention (needs assessment) and post-intervention (outcome evaluation) across TDF domains. *Post-intervention: *N* = 54. ^†^Post-intervention: *N* = 53. TDF, Theoretical Domains Framework; SDM, shared decision-making.

There was an overall increase in competencies, opportunities and motivations post-intervention, with significant differences found in most of the measured TDF domains as well as SDM (Figure 5). The largest changes in mean ranks before and after intervention were found with “beliefs about capabilities,” “reinforcement” and “skills.” For “beliefs about consequences,” “intentions,” “goal priority,” “memory,” “attention” and “negative effect,” there was no significant difference in mean ranks before and after intervention, suggesting that the intervention had no effect on these variables (Figure 5).

As the intervention significantly improved SDM and results on most TDF domains, a stepwise multiple regression was conducted to evaluate which TDF domains could predict SDM. A total of 17 TDF domains were included in the analysis as independent variables, with SDM as the dependent variable. By selecting the most relevant predictors for SDM, the stepwise multiple regression focused on the TDF domains that had measurable and significant effect on SDM. The variable “beliefs about capabilities” was the single most relevant predictor (Supplementary Table 4). This independent variable explained 23.7% of the variance in SDM in the pre-intervention period [*R*^2^ = 0.237, *F*(1, 43) = 13.355, *p* = 0.001] and 44.6% of the variance in SDM in the post-intervention period [*R*^2^ = 0.446, *F*(1, 48) = 38.610, *p* < 0.001]. The association between “beliefs about capabilities” and SDM was stronger post-intervention (*β* = 0.668, *p* < 0.001) than pre-intervention, further supporting the difference of 20.9 percentage points.

The intervention was found to significantly enhance both beliefs about capabilities, a key predictor of SDM, and SDM itself. Additionally, since beliefs about capabilities have a significant impact on SDM, improvements to the intervention to target these beliefs could further enhance SDM. We explore these possibilities in the Outlook and Future Research section, highlighting proven behavior change techniques that can influence such beliefs and strengthen the VR intervention.

## 4 Discussion

VR as a means for immersive learning is increasingly gaining attention in CME. A previous “lived experience” study, in which learners adhered to the daily regimen of a patient for 2 weeks, resulted in high dropout rates (23). In contrast to this, VR technology may offer a more feasible and realistic option for immersive experiences. The ability of VR to create impactful simulations is evidenced by its application in areas such as gender and racial discrimination (24). To the authors’ knowledge, ENGAGE is the first educational program to assess the impact of VR on HCP education and patient–HCP communication in MG, and to be published on these topics.

Our analysis, which integrated qualitative and quantitative insights, showed that the program was well received by HCPs. High satisfaction rates were reported for the learning content and technology in terms of ease of use, and an increase in the knowledge of, and attitude toward, MG symptoms and their impact on patients’ lives was observed. HCPs unanimously agreed to having gained more empathy for patients and a greater appreciation of social challenges and frustrations caused by MG symptoms. Empathy is an important aspect of patient-centered care (25), which in turn influences SDM (26, 27).

According to the TDF, strengthening behavioral determinants provides a credible mechanism through which training interventions can lead to sustained improvements in communication quality and patient-centered care (28). The improvements observed within TDF domains, such as “beliefs about capabilities,” “knowledge,” and “skills,” are particularly meaningful. These domains are immediate drivers of clinician behavior, and improved clinician behavior can directly translate into tangible benefits for patients. For instance, enhanced beliefs about capabilities contribute to greater clinician confidence in engaging patients in SDM, which is known to improve patient understanding, treatment adherence, and satisfaction (7, 29).

Among all TDF domains, “beliefs about capabilities” emerged as the most critical predictor of SDM, with a substantial increase in influence post-intervention. This underscores the critical role of self-efficacy in the SDM process. Self-efficacy, defined as “beliefs in one’s capabilities to organize and execute the courses of action required to produce given attainments,” is a key psychological construct that dynamically interacts with capabilities and behavior (30, 31). This construct contributes significantly to motivation and performance (31). It is important to highlight that self-efficacy is not a belief about someone’s ability to perform a behavior, but a self-perceived belief about their ability to manage challenging situations. Individuals with high self-efficacy are more likely to remain task-diagnostic and solution-focused (31, 32). Hence, our results indicate the importance of HCPs’ motivation and perception in their ability to implement SDM in difficult situations. It follows that building and sustaining HCPs’ self-efficacy beliefs is key for an intervention aiming to enhance SDM. By fostering a stronger sense of self-efficacy, HCPs could improve patient involvement in SDM, and in turn, improve patient-centered care.

Our results suggest that HCPs’ confidence in their ability to engage in and influence the decision-making process is paramount in determining the extent of their participation in SDM. The use of VR as a tool to increase HCPs’ confidence in SDM practices has been reported for HCPs involved in the care of pediatric patients (33). Our study demonstrates that this application of VR is also applicable to HCPs involved in the care of adults with MG.

Another important aspect related to the impact of an educational program is the concept of relevance. Previous studies have shown that “relevance” is a strong driver of satisfaction and knowledge gain, while commitment to change is fostered by the opportunity to apply the new knowledge (34). By including both patients and HCPs in the steering committee and needs assessment, we ensured that the content of the VR module reflected the lived experience of patients with MG and incorporated relevant symptoms and situations from their perspective. The VR module also underwent informal testing by four patients with MG during a standalone educational meeting, further ensuring content relevance. The VR experience facilitated an enhanced appreciation of the impact of certain MG symptoms among learners. Some HCP participants reported strong emotional reactions, including frustration, and indicated that the experience altered their perception of the relevance of symptoms. This was followed by an expressed intent to change their behavior in relation to communication skills, listening skills and showing empathy.

Our study confirmed the value of VR-based medical education as a tool to facilitate high levels of immersion for participants. The tool supported a better appreciation by HCPs of the impact of symptoms on a patient’s daily life, thus increasing the relevance as well as the recall of the learning experience. This effect may be especially relevant in the management of rare diseases, where HCPs within multidisciplinary teams may not see these patients on a regular basis, as well as for students and residents. In the authors’ opinion, VR-based immersive learning may be considered an excellent tool to overcome gaps in medical education, which is primarily built on curriculum-based textbook learning. As Chang et al. (35) recently reported, HCPs had better confidence in dealing with advanced medical decisions for patients following VR immersion; these changes were also noted 3 months after their VR exposure. This finding was supported by our program participants, who suggested using the VR experience to educate their multidisciplinary teams and residents on MG.

### 4.1 Outlook and future research

This exploratory pilot study highlighted the potential value of VR-based medical education in a rare disease. Indeed, this research in MG could be translated to other neuromuscular or rare diseases. However, data to reflect the patient experience and relevant symptoms in those specific diseases would be required.

The analysis provided insights that could enhance the impact of educational programs through behavior change techniques (BCTs) such as verbal persuasion, structured reflection and group discussion (21, 30, 36). Provision of structured feedback to HCPs about their SDM capabilities and specific examples of successful patient interactions could significantly enhance self-efficacy. To further strengthen self-efficacy beliefs, focusing on past successes could encourage HCPs to recognize their previous achievements in SDM. Activities such as participating in moderated reflection sessions or maintaining journals to document successful SDM encounters could facilitate this process. Group discussions and peer exchanges could promote positive social comparisons and serve as powerful motivational tools, further reinforcing self-efficacy and feelings of competence (21, 30, 36). Integrating these BCTs into the VR intervention could provide HCPs with structured opportunities to build confidence through targeted feedback and reflection on their past successes.

VR was chosen in place of HCP–patient interviews for this study by the steering committee based on the existing body of evidence demonstrating its power to increase empathy and provide impactful learning experiences. The learning impact of the VR-based intervention may be enhanced by using the tool in an optimal learning setting. For example, the addition of a peer-to-peer group discussion following completion of the VR-based immersive experience may stimulate the exchange of positive experiences. Further, it would elucidate best communication practices on what physicians should ask their patients and stimulate the motivation to change daily practice through collective peer-to-peer influence. The synergy of knowledge uptake, acquired communication skills, and encouraged motivation will ultimately lead to the desired behavioral change in SDM.

To develop a more comprehensive database and objective picture of the changes and impact achieved, it may be beneficial to include patients’ perspectives on the achieved changes in their HCPs’ behavior. This is especially important for programs that address SDM and patient–HCP communication. Additionally, a long-term follow-up to determine the sustainability of the indicated behavioral changes would be favorable. For future programs, it may also be advisable to highlight the importance and value of scientific evaluations, in order to increase the learners’ motivation to participate actively in the needs assessment as well as the outcome evaluation.

### 4.2 Limitations

De-identified participant data prevented paired analysis. As a result, pre- and post-intervention comparisons were based on independent sample tests following checks for normality. While statistically appropriate given the distributional characteristics and sample size, this limited strong individual-level causal inferences, as changes could not be directly attributed to the same respondents over time.

The changes identified in knowledge and attitudes, and the indicated intent to change, were based on subjective data provided directly after the educational activity through the self-assessment of participating HCPs. Additional studies to objectively evaluate the behavior of HCPs and improvement in SDM following an immersive educational experience would further enhance this research. In addition, despite significant efforts, only approximately two-thirds of HCPs who participated in the VR experience completed the outcome evaluation, limiting insights to a relatively small cohort. Further, it should be acknowledged that more than half of the HCP participants were neurologists or neuromuscular specialists. This reflects an opportunity for future work to trial the VR intervention across a wider spectrum of HCPs, thereby enhancing the generalizability of the findings. Finally, the program was only available in English, which may have negatively impacted participation in, and completion of, the surveys, as well as causing dropouts of non-native speakers (two HCPs) in the actual VR experience.

The availability of hardware and the costs associated with technical development of the VR software must also be considered, as currently, these may limit broader use of VR based educational programs in clinical practice. Moreover, the scalability of VR programs may be limited due to license-based models and private ownership. Through ownership of programs by CME providers, increased use of VR and wider platform-based availability, as seen with gaming technology, the costs of technical development and maintenance are likely to decrease. This could enable a broader rollout of VR-based programs, making them a more accessible and integral part of educational curricula for HCPs.

## 5 Conclusion

To our knowledge, ENGAGE is the first patient and HCP co-led, co-authored study in MG to evaluate the impact of a VR-assisted immersive learning experience on HCPs. Our study found that the immersive VR intervention was successful in increasing HCP empathy, knowledge and attitudes in the context of MG, suggesting that VR-based immersive learning can be a valuable tool for medical education and the improvement of SDM. Including the patient voice in the development of ENGAGE was a key success factor, as it ensured the relevance of the program’s content. Patient involvement should become an integral part of the design process of patient-centric programs, complemented by the inclusion of the patient voice. This educational project may serve as a model for future VR-based programs, encouraging further research on motivations, opportunities and barriers in patient–HCP interaction and assessing impact on behavioral change in MG and other diseases.

## Data Availability

The datasets presented in this article are not readily available because data from non-interventional studies are outside of UCB’s data sharing policy and are unavailable for sharing. Requests to access the datasets should be directed to Ivo Vlaev, vlaev@nus.edu.sg.

## Footnotes

1
www.qualtrics.com
