# Supplementary material for: ENGAGE: Analyzing the value of virtual reality in a patient-centric immersive learning program in myasthenia gravis for healthcare professionals
Source: Front Neurol. 2026 Jan 13;16:1655351. doi: 10.3389/fneur.2025.1655351 (PMC12836303; doi:10.3389/fneur.2025.1655351)
Supplement: Supplementary file 1 [file Table_1.pdf]

Supplementary Material

Contents

Supplementary Table 1. Qualitative insights from outcome evaluation interviews 2
Supplementary Table 2. Tests of normality 7
Supplementary Table 3. Mann–Whitney U test for testing differences pre-intervention (needs assessment) and post-intervention (outcome evaluation)* 8
Supplementary Table 4. Summary of stepwise multiple regression results to evaluate which TDF domains could predict SDM pre-intervention (needs assessment) and post-intervention (outcome evaluation) 9

Supplementary Table 1. Qualitative insights from outcome evaluation interviews

Construct	Clarification	Supporting quote(s)
**Satisfaction (Moore's Level 2)**	Overall experience of the learning activity	*"The concept of a story, which felt like it had a linear clear linear flow to […] it all felt like it was immersive, it felt like you were part of that environment.*"<br>*"I was incredibly awed. I mean, I was a little, I'm always a little sceptical. But no, I thought it was like, really, really well done. […] I heard nothing but positive comments. So awesome. I even heard some people that weren't able to make it because of other obligations were kind of disappointed they couldn't do it."*<br>*"I did not expect it to be that great."*<br>*"Yeah, really interesting. Overall, a really insightful experience. Definitely kind of captured what a patient might feel. For me, it really captured that frustration of trying to do a task or trying to, you know, move quicker […]"*
	Relevance of content	*"I liked the idea of it being a story […] put some context to it […] that was very good."*<br>*"I think some of the effects like you know, when you try to raise your arm and […] looking at your arm and not seeing moving, were quite an interesting experience. And I think, reminiscent probably of what a patient with MG would experience."*
	Ease of engagement with VR technology	*"[…] everyone got it. I mean, I was the oldest person that did it. So, you know, if anyone's gonna be technologically challenged, it would be me. And I think I got it pretty easily. And I don't think there were any concerns at all with technology."*<br>*"I thought it was pretty easy to use. I'm not very, like technologically savvy. […] I think it was very, like user friendly."*<br>*"[…] the interface was quite easy to get used to […] it was very clear that there were boundaries. So, in terms of user friendliness […] we didn't bump into each other... It was practical in that way."*<br>*"[T]he actual setup and using the VR, it was quite easy and quite tech minded. […] it was quite easy to set up, easy to navigate through."*

Construct	Clarification	Supporting quote(s)
	Effectiveness of VR as a learning tool	*“I've never used VR before, and I was impressed. […] you felt that you were experiencing it from the viewpoint of somebody else.”*<br>*“If the aim [of a VR experience] is to show people who do not have much experience with MG a little bit about what MG is, then that's far better than reading it in a textbook.”*<br>*“Once you experience what the patient is sensing yourself, you remember that – it makes an impression. You don’t remember what you read in a book, but you remember a VR experience.”*<br>*“I've just learned how amazing VR is. It's just like, wow, this is a whole new world. And actually, in this day of technology, using things like that, to help train other health professionals, is just yeah, I think it would be really, really good if that could be something that we can use […].”*
	Satisfaction with duration	*“I thought it was a good amount of time […] I don't think it was either too short or too long. I think it was perfect.”*<br>*“We felt that it went very quick. It wasn't too burdensome, and it was not boring.”*<br>*"It was about 20–25 minutes, which was definitely enough time to get immersed in it. It didn’t feel too long or too short."*
	Satisfaction with VR technology	*“[T]he graphics weren't as good as I've seen in video games. But that that's a minor issue.”*<br>*"The system needs to be a little bit more realistic – for example, allowing you to walk towards things rather than pressing a button."*
**Declarative knowledge (Moore’s Level 3a)**	Better understanding of breadth of MG symptoms	*“[…] it was really impactful to see what their day-to-day lives can be. Particularly striking for me was the difficulties they have with their vision, like as the ptosis worsens, and the diplopia worsens, and things like that. So, I think that was really insightful and kind of gives us like a tool to understand what they're going through to an extent.”*<br>*“So, I think, you know, interestingly, we had a new onset MG patient, maybe a month after the experience. So, she had never been diagnosed before and has like listening to her history and like taking it back, you know, she was like, well, retrospectively, now that I see that all of this weakness [was] happening. This happened like a few months ago, when I was willing to chalk it up to old age and, you know, all these other things.”*

Construct	Clarification	Supporting quote(s)
		"I guess just seeing it from a patient's perspective, you know, I like to think that I sort of understand what it's like, but actually, that was much more hard hitting than just a patient telling me what it's like having double vision, just seeing what it's actually like having double vision was a was a big thing."
	Better understanding of the impact of MG on a patient's life	*"I think it's hard to imagine what things like blurred vision and double vision feel like on a day-to-day basis, when you've never experienced those things in and of themselves. […] And so, I think the VR component of that […] was really it's helpful to see that because it's hard to imagine it."* *"I'd like to think that I understand it from a patient's point, but actually being able to really see what it looks like to have double vision is horrible."* *"You get this feeling of the patient's life. Yeah, yeah, glimpse into the patient's life."*
	Developing more empathy for patients with MG	*"[Empathy], yes, because you could actually experience the frustration and the difficulty of how you feel like if you're exposed to the symptom."* *"I think it breeds empathy […] [and] motivates us all to improve our communication skills."* *" […] the scene at work and a scene at home where her partner and her colleagues are, you know, kind of giving her a bit of a hard time, really, I think forced me to take a different perspective and empathize a little bit more with patients."* *"I think it definitely helps you empathize more with your patient group. And I definitely link in more with that frustration side, like it must be really annoying when they want to do a task, or they want […] and they just can't make it. And I think the VR experience really heightened that frustration element to me, because patients talk about it all the time. But I think unless you've experienced it, it's really difficult."*
	Developing an awareness of what patients with MG might fear	*"[…] it was really impactful to see what their day to day lives can be, particularly striking for me was […] the difficulties they have with their vision, like as the ptosis worsens, and the diplopia worsens, and things like that. So, I think that was really insightful and kind of gives us […] a tool to understand what they're going through to an extent."*

Construct	Clarification	Supporting quote(s)
	Understanding of what might be important in terms of treatment options	*"[…] in the treatment of MG, I think there's a level of complacency in what's been called treatment inertia. And I think that, you know, to give this perspective to more doctors, more nurse practitioners, people taking care of people with MG, I think it will hopefully push some people beyond that […]."*<br>*"I think it helps with the understanding, you know […] it gives you a better insight into the things that are important to patients and things that impact their day-to-day life […]. And so I think that that will make a big difference in coming to better shared decisions with patients."*
	What to do to better communicate with patients with MG	*"[…] I think that the doctor [in the VR module] showed, you know, pretty well, how not to communicate with patients […] So, I think that once we, as doctors, kind of see that it makes us question our own communication skills and really improve upon them."*<br>*"So, I think it would definitely help with not only communicating with patients, because you've got a better understanding. And again, the empathy is better. But I think that [it would help] communication within teams […]."*
**Procedural knowledge (Moore's Level 3b)**	Confidence knowing what matters to the patient	*"I like to think we're quite good at SDM […] in our team anyway. [But] making a bit more of an effort to understand what might interfere with their plans. And I just thought, you know, I hadn't really thought of that. You know, it's all very well, we might say, right, these are your treatment options. We'll give you the information, and we'll talk you through it. But that's something that I don't ever really consider about what you know, why is it that you're not making that decision?"*
	Confidence in better understanding the impact of MG on a patient's life	*"I think […] what it will make me do is really kind of reframe some of my questions. So, asking about the symptoms in the context of one's day-to-day life. So, I have a better understanding of that rather than you know, are you having double vision [and] are you having swallowing trouble?"*
	Know how the patient feels about the illness/its management	*"If someone's refusing or deciding not to, then maybe we need to explore it, rather than just take that as they don't want treatment, you know, and again, it might be that people just don't want treatment. And that's fine. But at least if we've explored it a little bit more as to why I think that's the bit that maybe we're not very good at is finding out why they might be resistant."*

Construct	Clarification	Supporting quote(s)
		"If they're not doing that [i.e. SDM] already that is a little bit embarrassing, but if they're not, this will tell them that they need to do that. They need to dig a little bit deeper into the patient's life and understand what the lived experience is with this disease."
**Competence gain and commitment to change (Moore's Level 4)**	Intention to change my practice	*"I'm a little bit more in tune with addressing each and every symptom and really trying to, you know, adjust my treatment on the basis of those symptoms and on the basis of what patients want, right? […] So, I think in that sense, […] it heightens my ability or my desire to do some SDM."*<br>*"I think just, yeah, just maybe a bit more empathy and […] trying to be a bit more patient and understanding. Yeah, how it really impacts on someone."*<br>*"[It] will make me a little bit more understanding rather than just, if a patient says 'No, I don't want treatment', to say 'Actually, why don't you? What's that all about? What's stopping you? Yeah, what are your concerns?' We've got a guy at the moment, who is a young guy who's been offered a thymectomy, [and] he's decided he doesn't want it [...] but we need to encourage him to have it, because actually, that's the best thing he can have […] None of us have actually really started picking apart 'Why doesn't he want it?' And actually, two weeks ago [after participating in the VR experience], I met with him and started having those conversations […] he's in denial, he's struggling to accept his diagnosis. He can't understand what the point of surgery is. So, it was just that actually. You know, I think we're quite good as clinicians […] we've involved them in that decision making, but if they don't want to do it that's their choice, and they can make that wrong decision, rather than trying to pick it apart a little bit more as to why you're doing it. And as a result, he's now turned around and said, 'Actually, I will have surgery'."*<br>*"[…] how [it] is going to change my practice is by recommending to people who are new to MG, who are in the training positions who are coming through the department. And then the nurses, perhaps even the managers who, who we are trying to convince that we need more investment in the team to develop from authentic patients. […] so training and educating non-specialists and the aligned health care professionals."*<br>*"How I will change my practice would be to see what I can do to acquire a couple of these goggles and then use it in my training. So, I do a lot of training and teaching for MG, and I would like to incorporate the actual experience of it."*

HCP, healthcare professional; MG, myasthenia gravis; SDM, shared decision-making; VR, virtual reality.

Supplementary Table 2. Tests of normality

Measurement		*Kolmogorov-Smirnov*[*]			*Shapiro-Wilk*		
		Statistic	df	Sig.	Statistic	df	Sig.
Pre (Needs assessment)	SDM	0.090	45	0.200[†]	0.976	45	0.484
	Knowledge	0.269	45	0.000	0.885	45	0.000
	Skills	0.296	45	0.000	0.829	45	0.000
	Identity	0.268	45	0.000	0.825	45	0.000
	Beliefs about capabilities	0.152	45	0.011	0.949	45	0.046
	Optimism	0.287	45	0.000	0.823	45	0.000
	Beliefs about consequences	0.242	45	0.000	0.805	45	0.000
	Reinforcement	0.238	45	0.000	0.860	45	0.000
	Intentions	0.245	45	0.000	0.867	45	0.000
	Goal frequency	0.310	45	0.000	0.829	45	0.000
	Goal priority	0.278	45	0.000	0.869	45	0.000
	Memory	0.268	45	0.000	0.845	45	0.000
	Attention	0.200	45	0.000	0.907	45	0.002
	Environmental context resources	0.187	45	0.000	0.947	45	0.040
	Social influences	0.194	45	0.000	0.889	45	0.000
	Positive affect	0.283	45	0.000	0.798	45	0.000
	Negative affect	0.209	45	0.000	0.880	45	0.000
	Behavioral regulation	0.245	45	0.000	0.924	45	0.006
Post (Outcome evaluation)	SDM	0.170	52	0.001	0.875	52	0.000
	Knowledge	0.249	52	0.000	0.857	52	0.000
	Skills	0.291	52	0.000	0.803	52	0.000
	Identity	0.283	52	0.000	0.717	52	0.000
	Beliefs about capabilities	0.221	52	0.000	0.888	52	0.000
	Optimism	0.269	52	0.000	0.845	52	0.000
	Beliefs about consequences	0.337	52	0.000	0.737	52	0.000
	Reinforcement	0.231	52	0.000	0.891	52	0.000
	Intentions	0.239	52	0.000	0.821	52	0.000
	Goal frequency	0.190	52	0.000	0.890	52	0.000
	Goal priority	0.258	52	0.000	0.889	52	0.000
	Memory	0.257	52	0.000	0.888	52	0.000
	Attention	0.239	52	0.000	0.889	52	0.000
	Environmental context resources	0.138	52	0.015	0.974	52	0.321
	Social influences	0.157	52	0.003	0.893	52	0.000
	Positive affect	0.280	52	0.000	0.842	52	0.000
	Negative affect	0.191	52	0.000	0.902	52	0.000
	Behavioral regulation	0.119	52	0.065	0.958	52	0.064

[*]Lilliefors significance correction. [†]This is a lower bound of the true significance.
df, degrees of freedom; SDM, shared decision-making; Sig., significance.

Supplementary Table 3. Mann–Whitney U test for testing differences pre-intervention (needs assessment) and post-intervention (outcome evaluation)*

	Mann–Whitney U	*Wilcoxon W*	*Z*	*Asymp. Sig. (2-tailed)*	*Monte Carlo Sig. (2-tailed)*		
					Sig.	99% CI	
						Lower bound	Upper bound
SDM	918.000	1953.000	−2.214	0.027	0.024†	0.020	0.028
Knowledge	718.500	1753.500	−3.671	0.000	0.000†	0.000	0.001
Skills	684.500	1719.500	−4.189	0.000	0.000†	0.000	0.000
Identity	972.000	2007.000	−1.995	0.046	0.048†	0.042	0.053
Beliefs about capabilities	564.500	1599.500	−4.735	0.000	0.000†	0.000	0.000
Optimism	967.000	2002.000	−2.044	0.041	0.039†	0.034	0.044
Beliefs about consequences	970.000	2005.000	−1.736	0.083	0.085†	0.078	0.092
Reinforcement	628.500	1663.500	−4.426	0.000	0.000†	0.000	0.000
Intentions	1083.000	2118.000	−1.125	0.261	0.268†	0.256	0.279
Goal frequency	835.000	1870.000	−2.980	0.003	0.002†	0.001	0.004
Goal priority	1002.500	2037.500	−1.758	0.079	0.079†	0.072	0.086
Memory	1102.000	2137.000	−0.991	0.322	0.328†	0.316	0.340
Attention	1004.500	2039.500	−1.684	0.092	0.098†	0.090	0.105
Environmental context resources	797.500	1832.500	−3.098	0.002	0.002†	0.001	0.003
Social influences	891.000	1926.000	−2.434	0.015	0.014†	0.011	0.017
Positive affect	878.000	1913.000	−2.726	0.006	0.006†	0.004	0.008
Negative affect	1014.000	2049.000	−1.606	0.108	0.110†	0.102	0.118
Behavioral regulation	741.500	1776.500	−3.507	0.000	0.001†	0.000	0.001

*Grouping variable: Measurement. †Based on 10,000 sampled tables with starting seed 2,000,000. Asymp., asymptotic; CI, confidence interval; SDM, shared decision-making; Sig., significance.

Supplementary Table 4. Summary of stepwise multiple regression results to evaluate which TDF domains could predict SDM pre-intervention (needs assessment) and post-intervention (outcome evaluation)

	Needs assessment	*Outcome evaluation*
Observations, N	44	49
R	0.487*	0.668*
R square	0.237	0.446
Adjusted R square	0.219	0.434
Standard error of the estimate	1.10344	1.06034
F	13.355	38.610
Significance	0.001†	<0.001‡

*Dependent variable: SDM. †Significant predictors: (Constant) "beliefs about capabilities" (β=0.487, p=0.001). ‡Significant predictors: (Constant) "beliefs about capabilities" (β=0.668, p<0.001). TDF, Theoretical Domains Framework; SDM, shared decision-making.
